# Independent association of history of diabetic foot with all-cause mortality in patients with type 2 diabetes: the Renal Insufficiency And Cardiovascular Events (RIACE) Italian Multicenter Study

**DOI:** 10.1186/s12933-023-02107-9

**Published:** 2024-01-13

**Authors:** Martina Vitale, Emanuela Orsi, Anna Solini, Monia Garofolo, Veronica Resi, Enzo Bonora, Cecilia Fondelli, Roberto Trevisan, Monica Vedovato, Giuseppe Penno, Giuseppe Pugliese

**Affiliations:** 1 Department of Clinical and Molecular Medicine, “La Sapienza” University, Via di Grottarossa, Rome, 1035-1039 - 00189 Italy; 2 Diabetes Unit, Fondazione IRCCS “Cà Granda - Ospedale Maggiore Policlinico”, Milan, Italy; 3 Department of Surgical, Medical, Molecular and Critical Area Pathology, University of Pisa, Pisa, Italy; 4 Department of Clinical and Experimental Medicine, University of Pisa, Pisa, Italy; 5 Division of Endocrinology, Diabetes and Metabolism, University and Hospital Trust of Verona, Verona, Italy; 6 Diabetes Unit, University of Siena, Siena, Italy; 7 Endocrinology and Diabetes Unit, Azienda Ospedaliera Papa Giovanni XXIII, Bergamo, Italy; 8 Department of Clinical and Experimental Medicine, University of Padua, Padua, Italy

**Keywords:** Type 2 diabetes, All-cause mortality, Diabetic foot, Foot ulcer, Amputation, Lower limb revascularization

## Abstract

**Background:**

Foot ulcers and/or infections are common long-term complications of diabetes and are associated with increased mortality, especially from cardiovascular disease, though only a few studies have investigated the independent contribution of these events to risk of death. This study aimed at assessing the association of history of diabetic foot with all-cause mortality in individuals with type 2 diabetes, independent of cardiovascular risk factors, other complications, and comorbidities.

**Methods:**

This prospective cohort study enrolled 15,773 Caucasian patients in 19 Italian centers in the years 2006–2008. Prior lower extremity, coronary, and cerebrovascular events and major comorbidities were ascertained by medical records, diabetic retinopathy by fundoscopy, diabetic kidney disease by albuminuria and estimated glomerular filtration rate, cardiovascular risk factors by standard methods. All-cause mortality was retrieved for 15,656 patients on 31 October 2015.

**Results:**

At baseline, 892 patients (5.7%) had a history of diabetic foot, including ulcer/gangrene and/or amputation (n = 565; 3.58%), with (n = 126; 0.80%) or without (n = 439; 2.78%) lower limb revascularization, and revascularization alone (n = 330; 2.09%). History of diabetic foot was associated with all-cause death over a 7.42-year follow-up (adjusted hazard ratio, 1.502 [95% confidence interval, 1.346–1.676], *p* < 0.0001), independent of confounders, among which age, male sex, smoking, hemoglobin A_1c_, current treatments, other complications, comorbidities and, inversely, physical activity level and total and HDL cholesterol were correlated independently with mortality. Both ulcer/gangrene and amputation alone were independently associated with death, with a higher strength of association for amputation than for ulcer/gangrene (1.874 [1.144–3.070], *p* = 0.013 vs. 1.567 [1.353–1.814], *p* < 0.0001). Both ulcer/gangrene/amputation and lower limb revascularization alone were independently associated with death; mortality risk was much higher for ulcer/gangrene/amputation than for revascularization (1.641 [1.420–1.895], *p* < 0.0001 vs. 1.229 [1.024–1.475], *p* = 0.018) and further increased only slightly for combined ulcer/gangrene/amputation and revascularization (1.733 [1.368–2.196], *p* < 0.0001).

**Conclusions:**

In patients with type 2 diabetes, an history of diabetic foot event, including ulcer/gangrene, amputation, and lower limb revascularization, was associated with a ~ 50% increased risk of subsequent death, independent of cardiovascular risk factors, other complications and severe comorbidities, which were also significantly associated with mortality. The association with mortality was greatest for amputation, whereas that for revascularization alone was relatively modest.

**Trial registration:**

ClinicalTrials.gov, NCT00715481, retrospectively registered 15 July, 2008.

**Supplementary Information:**

The online version contains supplementary material available at 10.1186/s12933-023-02107-9.

## Background

Diabetic foot ulcers are serious sequelae of diabetes that occur in 19 to 34% of patients during their lifetime [1], with recurrence rates of ~ 65% at 3–5 years [2]. Ulcers are commonly classified by etiology into ischemic, neuropathic, or neuro-ischemic, depending on whether they result from peripheral artery disease (PAD), diabetic peripheral neuropathy (DPN), or both, with PAD and DPN also causing gangrene and Charcot arthropathy, respectively, which are frequently associated with foot ulcers [2]. In addition, ~ 50% of ulcers are complicated by superimposed infection [3], though foot infections involving soft tissue or bone can rarely have an hematogenous origin and develop in the absence of an ulcer [4]. All these manifestations of the so-called “diabetic foot” may require minor or major amputations, with an estimated lifetime incidence of 20% [2]. Data from multiple sources showed a 51% decline in the incidence of lower limb amputations among US adults from 1990 to 2010 [5], though a more recent survey indicated a resurgence in this and other diabetic complications between 2010 and 2015, especially in younger individuals [6].

Diabetic foot ulcers are associated with high morbidity and mortality, especially from cardiovascular disease (CVD) [7–10], with a pooled relative risk of 2.45, as reported in a recent meta-analysis [11]. Death rates were shown to be ~ 45% at 5 years and ~ 70% at 10 years [2, 12–14], higher for ischemic (and neuro-ischemic) than for neuropathic ulcers [15–17], and with an apparently declining trend [16, 18, 19], which however was not consistently observed [13]. The high mortality rates associated with foot ulcers (and the diabetic foot in general) have been attributed to the coexistence of other micro and macrovascular complications of the disease [7]. In fact, CVD in the lower limb and other vascular beds as well as chronic kidney disease or dialysis were found to be independent risk factors for mortality, together with age, male sex, smoking habits, long diabetes duration, high HbA_1c_ and low hemoglobin and albumin levels [13, 16–26]. Risk of death was also related to ulcer duration [18], recurrence [19, 23], severity [24], and need for amputation [17, 21, 22]. However, only a few studies assessed the impact of foot ulcer per se, showing that it remained significantly associated with death after adjusting for confounders, suggesting a direct and independent relationship with mortality [20, 25, 26]. However, these surveys provided discordant figures, which may reflect differences in the national health systems, settings, and time-periods.

The present analysis aimed at assessing the extent of association of history of diabetic foot, including but not limited to foot ulcers, with all-cause mortality in patients with type 2 diabetes, independent of CVD risk factors, other complications and comorbidities.

## Methods

### Design

The Renal Insufficiency And Cardiovascular Events (RIACE) Italian Multicenter Study was an observational, prospective, cohort study on the impact of estimated glomerular filtration rate (eGFR) on morbidity and mortality in individuals with type 2 diabetes [27]. The study was conducted in accordance with the Declaration of Helsinki. The study protocol was approved by the locally appointed ethics committees and participants gave informed consent.

### Patients

The RIACE study enrolled 15,933 Caucasian patients with type 2 diabetes, consecutively attending 19 hospital-based, tertiary referral Diabetes Clinics of the National Health Service throughout Italy, most of them in the years 2006–2008 (first patients 6 October 2005 - last patient 17 December 2008). Exclusion criteria were dialysis or renal transplantation. As 160 patients (1.0%) were excluded due to missing or implausible values, the study population consisted of the remaining 15,773 individuals.

### Baseline data

Baseline data were collected using a standardized protocol across participating centers [27].

Participants underwent a structured interview to collect the following information: age at the time of the interview, smoking status, physical activity (PA) level, known diabetes duration, severe co-morbidities, and current glucose-, lipid-, and blood pressure (BP)-lowering treatments. Patients were categorized by smoking status as never, former, or current smokers and by moderate-to-vigorous PA level as physically inactive or moderately inactive (< 60 min·week^− 1^), moderately active (60–150 min·week^− 1^), or highly active (> 150 min·week^− 1^). Comorbidities included chronic obstructive pulmonary disease (COPD), chronic liver disease, and cancer.

Body mass index (BMI) was calculated from weight and height, whereas waist circumference was estimated from log-transformed BMI values. Then, BP was measured with a sphygmomanometer with the patients seated with the arm at the heart level.

Hemoglobin A_1c_ (HbA_1c_) was measured by HPLC using DCCT-aligned methods; triglycerides and total and HDL cholesterol were determined in fasting blood samples by standard colorimetric enzymatic methods. Then, non-HDL cholesterol level was computed by subtracting HDL cholesterol from total cholesterol, whereas LDL cholesterol concentration was estimated using the Friedewald formula, i.e., LDL cholesterol = total cholesterol – HDL cholesterol – (triglycerides/5) (in mg/dl).

Previous major adverse CVD events, including myocardial infarction, stroke, foot ulcer/gangrene, non-traumatic amputation, and cerebrovascular, carotid, and lower limb revascularization, were adjudicated based on hospital discharge records by an ad hoc committee in each center [28]. Manifestations of diabetic foot included ulcer/gangrene, amputation, and lower limb revascularization, either alone or in combination. Amputation was defined as minor or major depending on whether it was below or above the ankle joint, respectively. Lower limb revascularization procedures were classified as endovascular or surgical. While lower limb revascularization was considered a CVD event, as myocardial infarction, stroke, and coronary or carotid revascularization, the cause of foot ulcer/gangrene and amputation could not be established, as no information was available regarding the presence and severity of PAD (except for revascularization), DPN, and/or foot infection in the RIACE participants. However, foot ulcer/gangrene were hypothesized to be ischemic (or neuro-ischemic) if occurred in individuals who underwent a lower limb revascularization procedure and amputation was hypothesized to be caused by infection if occurred in patients without ulcer/gangrene. In contrast, no assumption could be made regarding the etiology of ulcer/gangrene in patients who were not revascularized, as this does not necessarily imply a neuropathic origin, as well as of amputation in those with ulcer/gangrene, as it was impossible to establish the role of infection versus that of PAD in guiding decision to proceed with surgery.

The presence of diabetic kidney disease (DKD) was assessed by measuring albuminuria and serum creatinine, as previously detailed [27, 29]. Briefly, albumin excretion rate was obtained from 24-hour urine collections or calculated from albumin-to-creatinine ratio in early-morning, first-voided urine samples; albumin concentration in urines was measured by immunonephelometry or immunoturbidimetry, in the absence of interfering clinical conditions. Serum (and urine) creatinine was measured by the modified Jaffe method, traceable to IDMS, and eGFR was calculated by the 2009 Chronic Kidney Disease Epidemiology Collaboration equation [30]. Patients were then assigned to one of the following DKD phenotypes [31]: no DKD, albuminuria alone (albuminuric DKD with preserved eGFR), reduced eGFR alone (non-albuminuric DKD), or both albuminuria and reduced eGFR (albuminuric DKD with reduced eGFR).

The presence of diabetic retinopathy (DR) was assessed in each center by an expert ophthalmologist by dilated fundoscopy [32]. On the basis of the actual fundus appearance or the retinal disease condition that had eventually required previous photocoagulation or surgical treatment, patients were graded into the following categories: no DR; mild, moderate, or severe non-proliferative DR; proliferative DR; or diabetic macular edema. Patients with mild or moderate non-proliferative DR were classified as having non-advanced DR, whereas those with severe non-proliferative DR, proliferative DR, or diabetic macular edema were grouped into the advanced, sight threatening DR category. DR grade was assigned based on the worse eye.

### All-cause mortality

The vital status of study participants on 31 October 2015 was verified by interrogating the Italian Health Card database (http://sistemats1.sanita.finanze.it/wps/portal/), which provides updated and reliable information on all current Italian residents [33].

### Statistical analysis

Data are expressed as mean ± SD or median (interquartile range) for continuous variables, and number of cases and percentage for categorical variables. Patients were stratified by absence or presence of (a) history of diabetic foot event; (b) ulcer/gangrene and/or amputation; and (c) ulcer/gangrene/amputation and/or lower limb revascularization. Comparisons among the above categories were performed by unpaired Student’s t test or one-way ANOVA or Kruskal-Wallis test, according to the parametric or non-parametric distribution of continuous variables, and Pearson’s χ^2^ test, for categorical variables. Binary non-conditional multivariable logistic regression analysis with backward stepwise selection of variables was applied to assess the independent correlates of previous manifestations of diabetic foot; covariates were age, sex, smoking status, PA level, diabetes duration, HbA_1c_, BMI, triglycerides, total and HDL cholesterol, systolic and diastolic BP, anti-hyperglycemic, lipid-lowering, and anti-hypertensive therapy, DKD phenotype, DR grade, any coronary and cerebrovascular event, and any comorbidity. Data are presented as odds ratios (ORs) and their 95% Cis.

Crude mortality rates were described as events per 1,000 patient-years from start of follow-up to censoring, with 95% exact Poisson confidence intervals (CIs) and adjusted for age and sex by a Poisson regression model. Kaplan-Meier survival probabilities for all-cause mortality were estimated according to the above categories and differences were analyzed using the log-rank statistic. The hazard ratios (HRs) and their 95% CIs were estimated by Cox proportional hazards regression with backward selection of variables. These analyses were sequentially adjusted for age and sex (model 1), plus other CVD risk factors, i.e., smoking status, PA level, diabetes duration, HbA_1c_, BMI, triglycerides, total and HDL cholesterol, systolic and diastolic BP, and anti-hyperglycemic, lipid-lowering, and anti-hypertensive therapy (model 2), and plus presence of other complications (DKD, DR, and any coronary and cerebrovascular event), and any severe comorbidity (model 3).

All *p* values were two-sided, and a *p* < 0.05 was considered statistically significant. Statistical analyses were performed using SPSS version 13.0 (SPSS Inc., Chicago, IL, USA).

## Results

### History of diabetic foot at baseline

At baseline, 895 patients (5.7%) had a history of any diabetic foot event, 431 of them (48.3%) more than one. Of these individuals, 565 (3.58%) had ulcer/gangrene (412, 2.61%), amputation (33, 0.21%), or both (120, 0.76%); 126 (0.80%) of them were revascularized (69 with endovascular, 48 with surgical, and 9 with both procedures), whereas 439 (2.78%) were not. The remaining 330 patients (2.09%) underwent lower limb revascularization (123 with endovascular, 197 with surgical, and 10 with both procedures) without any ulcer/gangrene and/or amputation.

As shown in Table 1, patients with any foot event were older and more frequently males and former or current smokers, as compared with those without. In addition, they had longer diabetes duration, higher levels of HbA_1c_, triglycerides, TG:HDL ratio, systolic BP, pulse pressure, and albuminuria, and higher prevalence of dyslipidemia, hypertension, insulin, lipid-lowering, anti-hypertensive, anti-platelet, and anti-coagulant treatment, albuminuric and non-albuminuric DKD, non-advanced and advanced DR, any coronary and cerebrovascular event, including myocardial infarction, stroke, and revascularization procedures, COPD, and chronic liver disease. In contrast, they had lower levels of total, HDL, non-HDL, and LDL cholesterol, diastolic BP, and eGFR as well as lower prevalence of cancer.

**Table 1:** Baseline clinical features of study participants by history of diabetic foot

Variables	History of diabetic foot		*P*
	No	Yes	
N (%)	14,878 (94.3)	895 (5.7)	
Ulcer/gangrene	–	532 (59.4)	
Amputation	–	153 (17.1)	
Minor	–	129 (14.4)	
Major	–	24 (2.7)	
Ulcer/gangrene/amputation	–	565 (63.1)	
Lower limb revascularization	–	456 (50.9)	
Endovascular	–	192 (21.5)	
Surgical	–	245 (27.4)	
Both	–	19 (2.1)	
Age, years	66.4 ± 10.4	70.1 ± 9.6	< 0.0001
Sex, n (%)			< 0.0001
Females	6,522 (43.8)	292 (32.6)	
Males	8,356 (56.2)	603 (67.4)	
Smoking, n (%)			< 0.0001
Never	8,511 (57.2)	417 (46.6)	
Former	4,101 (27.6)	333 (37.2)	
Current	2,266 (15.2)	145 (16.2)	
PA level, n (%)			< 0.0001
Inactive or moderately inactive	9,314 (62.6)	712 (79.6)	
Moderately active	5,333 (35.8)	178 (19.9)	
Highly active	231 (1.6)	5 (0.6)	
Diabetes duration, years	12.8 ± 10.0	19.2 ± 10.6	< 0.0001
HbA_1c_, %	7.53 ± 1.50	7.92 ± 1.59	< 0.0001
BMI, kg·m^− 2^	29.0 ± 5.2	28.8 ± 5.0	0.351
Waist circumference, cm	102.5 ± 11.1	102.9 ± 11.3	0.210
Triglycerides, mmol·l^− 1^	1.33 (0.97–1.88)	1.39 (1.02–2.01)	0.031
Total cholesterol, mmol·l^− 1^	4.80 ± 0.99	4.59 ± 1.01	< 0.0001
HDL cholesterol, mmol·l^− 1^	1.30 ± 0.35	1.20 ± 0.36	< 0.0001
Non-HDL cholesterol, mmol·l^− 1^	3.50 ± 0.95	3.36 ± 0.94	< 0.0001
LDL cholesterol, mmol·l^− 1^	2.80 ± 0.84	2.63 ± 0.85	< 0.0001
Dyslipidemia, n (%)	12,214 (82.1)	746 (83.4)	0.340
Systolic BP, mmHg	138.0 ± 17.9	139.3 ± 19.7	0.039
Diastolic BP, mmHg	78.8 ± 9.4	77.4 ± 9.9	< 0.0001
Pulse pressure, mmHg	59.1 ± 15.6	61.9 ± 16.9	< 0.0001
Hypertension, n (%)	12,369 (83.1)	820 (91.6)	< 0.0001
Anti-hyperglycemic treatment, n (%)			< 0.0001
Lifestyle	2,076 (14.0)	50 (5.6)	
Non-insulin	9,229 (62.0)	452 (50.5)	
Insulin	3,573 (24.0)	393 (43.9)	
Lipid-lowering treatment, n (%)	6,746 (45.3)	540 (60.3)	< 0.0001
Anti-hypertensive treatment, n (%)	10,398 (69.9)	751 (83.9)	< 0.0001
Albuminuria, mg·day^− 1^	13.1 (6.5–31.0)	25.6 (10.5–100.0)	< 0.0001
Serum creatinine, µmol·l^− 1^	80.1 ± 33.3	97.4 ± 47.1	< 0.0001
eGFR, ml·min^− 1^·1.73 m^− 2^	80.9 ± 20.7	69.4 ± 22.7	< 0.0001
DKD phenotype, n (%)			< 0.0001
No DKD	9,697 (65.2)	350 (39.1)	
Albuminuric DKD with preserved eGFR	2,767 (18.6)	228 (25.5)	
Non-albuminuric DKD	1,366 (9.2)	125 (14.0)	
Albuminuric DKD with reduced eGFR	1,048 (7.0)	192 (21.5)	
DR, n (%)			< 0.0001
No DR	11,797 (79.3)	479 (53.5)	
Non-advanced DR	1,758 (11.8)	199 (22.2)	
Advanced DR	1,323 (8.9)	217 (24.2)	
CVD, n (%)			
Myocardial infarction	1,503 (10.1)	255 (28.5)	< 0.0001
Coronary revascularization	1,246 (8.4)	338 (37.8)	< 0.0001
Any coronary event	2,020 (13.6)	395 (44.1)	< 0.0001
Stroke	463 (3.1)	52 (5.8)	< 0.0001
Carotid revascularization	589 (4.0)	278 (31.1	< 0.0001
Any cerebrovascular event	1,001 (6.7)	304 (34.0)	< 0.0001
Any coronary or cerebrovascular event	2,715 (18.4)	460 (52.1)	< 0.0001
Comorbidities n (%)			
Any	2,614 (17.6)	189 (21.1)	0.007
COPD	621 (4.2)	57 (6.4)	0.002
Chronic liver disease	1,264 (8.5)	106 (11.8)	0.001
Cancer	990 (6.7)	45 (5.0)	0.056

PA = physical activity; HbA_1c_ = hemoglobin A_1c_; BMI = body mass index; BP = blood pressure; eGFR = estimated glomerular filtration rate; DKD = diabetic kidney disease; DR = diabetic retinopathy; = CVD = cardiovascular disease; COPD = chronic obstructive pulmonary disease

The baseline clinical features of participants stratified by history of ulcer/gangrene and/or amputation are shown in Additional file 2: Table S1, whereas those of participants stratified by history of ulcer/gangrene/amputation and/or lower limb revascularization are shown in Additional file 3: Table S2.

History of diabetic foot was independently associated with age, male sex, smoking, diabetes duration, anti-hyperglycemic and lipid-lowering treatment, DKD, DR, any coronary and cerebrovascular event and, inversely, PA level and HDL cholesterol (Additional file 4: Table S3).

### Association between history of diabetic foot at baseline and all-cause mortality

As previously reported, valid information on vital status was retrieved for 15,656 participants (99.3% of the cohort). Of these individuals, 12,054 (76.99%) were alive, whereas 3,602 (23.01%) had deceased (follow-up duration: 7.42 ± 2.05 years, range 0–10.07; death rate: 31.02 per 1,000 person-years) [31, 34].

**Fig. 1:**
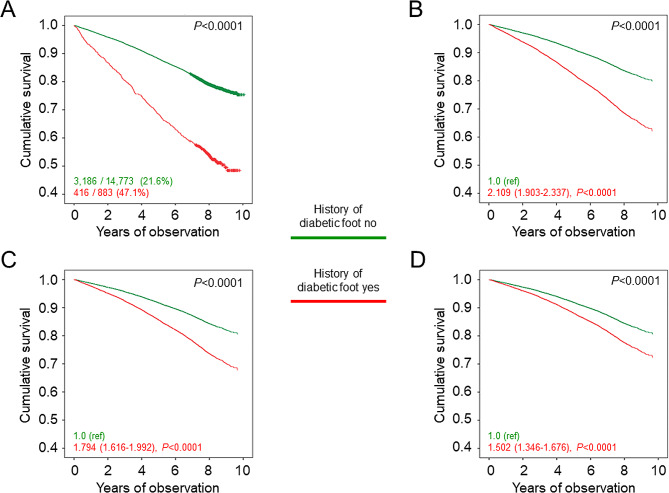
Survival analysis by presence or absence of history of diabetic foot. Kaplan Meier analysis (**A**) and Cox proportional hazards regression, adjusted for age and sex (model 1, **B**), plus smoking status, PA level, diabetes duration, HbA_1c_, BMI, triglycerides, total and HDL cholesterol, systolic and diastolic BP, and anti-hyperglycemic, lipid-lowering, and anti-hypertensive therapy (model 2, **C**), plus presence of other complications (DKD phenotypes, DR grades, any coronary and cerebrovascular event) and severe comorbidities (COPD, chronic liver disease, cancer) (model 3, **D**). Numbers (percentages) of deaths and HRs (95% CI) for mortality are shown for each group. PA = physical activity; HbA_1c_ = hemoglobin A_1c_; BMI = body mass index; BP = blood pressure; DKD = diabetic kidney disease; DR = diabetic retinopathy; COPD = chronic obstructive pulmonary disease; HR = hazard ratio; CI = confidence interval

**Fig. 2:**
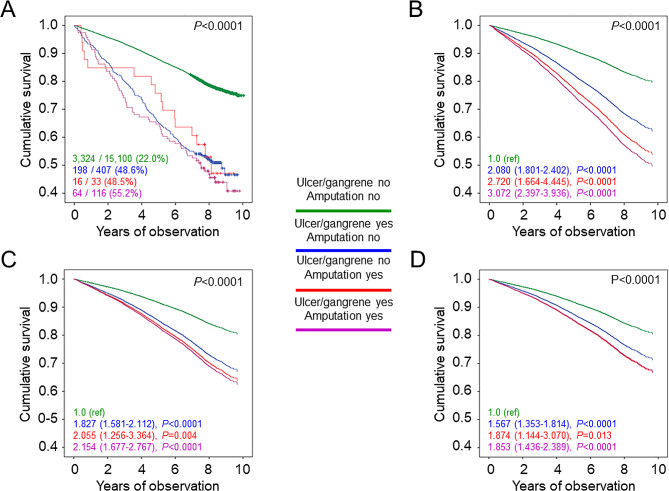
Survival analysis by presence or absence of history of ulcer/gangrene and/or amputation. Kaplan Meier analysis (**A**) and Cox proportional hazards regression, adjusted for age and sex (model 1, **B**), plus smoking status, PA level, diabetes duration, HbA_1c_, BMI, triglycerides, total and HDL cholesterol, systolic and diastolic BP, and anti-hyperglycemic, lipid-lowering, and anti-hypertensive therapy (model 2, **C**), plus presence of other complications (DKD phenotypes, DR grades, any coronary and cerebrovascular event) and severe comorbidities (COPD, chronic liver disease, cancer) (model 3, **D**). Numbers (percentages) of deaths and HRs (95% CI) for mortality are shown for each group. PA = physical activity; HbA_1c_ = hemoglobin A_1c_; BMI = body mass index; BP = blood pressure; DKD = diabetic kidney disease; DR = diabetic retinopathy; COPD = chronic obstructive pulmonary disease; HR = hazard ratio; CI = confidence interval

**Fig. 3:**
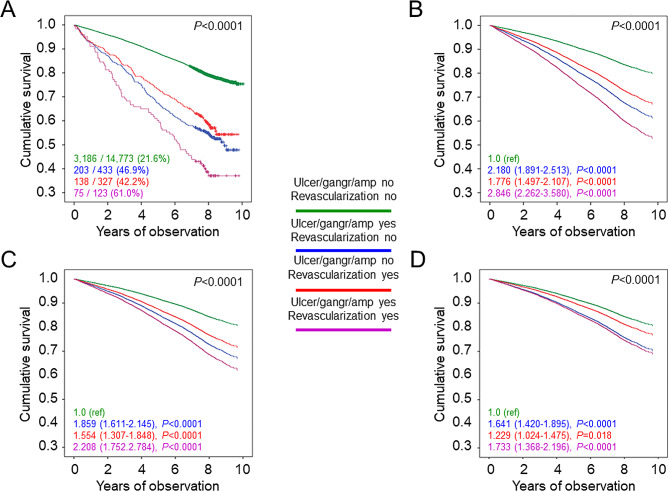
Survival analysis by presence or absence of history of ulcer/gangrene/amputation and/or lower limb revascularization. Kaplan Meier analysis (**A**) and Cox proportional hazards regression, adjusted for age and sex (model 1, **B**), plus smoking status, PA level, diabetes duration, HbA_1c_, BMI, triglycerides, total and HDL cholesterol, systolic and diastolic BP, and anti-hyperglycemic, lipid-lowering, and anti-hypertensive therapy (model 2, **C**), plus presence of other complications (DKD phenotypes, DR grades, any coronary and cerebrovascular event) and severe comorbidities (COPD, chronic liver disease, cancer) (model 3, **D**). Numbers (percentages) of deaths and HRs (95% CI) for mortality are shown for each group. PA = physical activity; HbA_1c_ = hemoglobin A_1c_; BMI = body mass index; BP = blood pressure; DKD = diabetic kidney disease; DR = diabetic retinopathy; COPD = chronic obstructive pulmonary disease; HR = hazard ratio; CI = confidence interval

As shown in Table 2, unadjusted death rate was markedly higher in patients with than in those without a history of diabetic foot event and remained 2-fold higher after adjustment for age and sex. Moreover, age- and sex-adjusted death rates were higher in patients with amputation vs. those with ulcer/gangrene alone and in patients with ulcer/gangrene/amputation vs. those with lower limb revascularization alone, with the highest rates observed in participants with both ulcer/gangrene and amputation and in those with both ulcer/gangrene/amputation and lower limb revascularization. Likewise, the Kaplan-Meier curves (Figs. 1A and 2A, and 3A) and unadjusted Cox proportional hazards regression showed an increased mortality in patients with any diabetic foot event (Table 3), ulcer/gangrene and/or amputation, and ulcer/gangrene/amputation and/or lower limb revascularization (not shown). When sequentially adjusting for confounders, risk of death remained ~ 50% higher in participants with any diabetic foot event (Fig. 1B–D; Table 3). In addition, it was higher in those with amputation with or without ulcer/gangrene than in those with ulcer/gangrene alone (Fig. 2B–D) and in those with ulcer/gangrene/amputation with or without lower limb revascularization than in those with lower limb revascularization alone, who only showed a ~ 23% increase in the adjusted mortality risk (Fig. 3B–D).

**Table 2:** Mortality rates in study participants by history of diabetic foot, ulcer/gangrene and/or amputation, and ulcer/gangrene/amputation and/or lower limb revascularization

	N	Events	Percent events	Events per 1,000 patient-years (95% CI), unadjusted	*P*	Events per 1,000 patient-years (95% CI), age- & sex-adjusted	*p*
History of diabetic foot no	14,773	3,186	21.6	28.78 (27.80–29.80)	Ref.	12.50 (11.10-14.09)	Ref.
History of diabetic foot yes	883	416	47.1	76.83 (69.79–84.58)	< 0.0001	25.51 (21.84–29.79)	< 0.0001
Ulcer/gangrene no – Amputation no	15,100	3,324	22.0	29.47 (28.49–30.49)	Ref.	12.53 (11.12–14.12)	Ref.
Ulcer/gangrene yes – Amputation no	407	198	48.6	81.27 (70.70-93.42)	< 0.0001	25.05 (20.87–30.07)	< 0.0001
Ulcer/gangrene no – Amputation yes	33	16	48.5	77.46 (47.45-126.43)	0.013	32.48 (19.59–53.84)	0.014
Ulcer/gangrene yes – Amputation yes	116	64	55.2	94.01 (73.58-120.11)	< 0.0001	36.89 (27.96–486.7)	< 0.0001
Ulcer/gangrene/amput no – Revasc no	14,773	3,186	21.6	28.78 (2780 − 29.80)	Ref.	12.50 (11.10-14.09)	Ref.
Ulcer/gangrene/amput yes – Revasc no	433	203	46.9	76.23 (66.43–87.47)	< 0.0001	26.31 (21.95–31.54)	< 0.0001
Ulcer/gangrene/amput no – Revasc yes	327	138	42.2	66.01 (55.87-78.00)	< 0.0001	21.74 (17.65–26.78)	< 0.0001
Ulcer/gangrene/amput yes – Revasc yes	123	75	61.0	113.50 (90.51-142.33)	< 0.0001	33.46 (25.80-43.39)	< 0.0001

CI = confidence interval

**Table 3:** Survival analysis by Cox proportional hazards regression with backward selection of variables, unadjusted and adjusted for confounders (model 3)

Variables	HR	95% CI	*p*
Any diabetic foot event (unadjusted)	2.73	2.46–3.02	< 0.0001
Any diabetic foot event	1.50	1.35–1.68	< 0.0001
Age, years	1.09	1.08–1.09	< 0.0001
Male sex	1.30	1.20–1.40	< 0.0001
Smoking status	–	–	0.001
Never	1.00	–	–
Former	1.07	0.99–1.16	0.074
Current	1.22	1.10–1.35	< 0.0001
PA level	–	–	0.010
Inactive or moderately inactive	1.00	–	–
Moderately active	0.90	0.83–0.97	0.004
Highly active	0.81	0.56–1.19	0.290
Diabetes duration, years	–	–	–
HbA_1c_, %	1.06	1.03–1.08	< 0.0001
BMI, kg·m^− 2^	–	–	–
Triglycerides, mmol·l^− 1^	1.00	1.00-1.01	0.092
Total cholesterol, mmol·l^− 1^	0.94	0.90–0.97	0.001
HDL cholesterol, mmol·l^− 1^	0.84	0.75–0.94	0.002
Systolic BP, mmHg	–	–	–
Diastolic BP, mmHg	0.99	0.99-1.00	< 0.0001
Anti-hyperglycemic treatment	–	–	< 0.0001
Lifestyle	1.00	–	–
Non-insulin	1.31	1.15–1.49	< 0.0001
Insulin	1.83	1.60–2.10	< 0.0001
Lipid-lowering treatment	0.78	0.73–0.84	< 0.0001
Anti-hypertensive treatment	1.18	1.08–1.29	< 0.0001
DKD phenotype	–	–	< 0.0001
No DKD	1.00	–	–
Albuminuric DKD with preserved eGFR	1.43	1.31–1.56	< 0.0001
Nonalbuminuric DKD	1.51	1.36–1.67	< 0.0001
Albuminuric DKD with reduced eGFR	1.89	1.71–2.09	< 0.0001
DR grade	–	–	0.001
No DR	1.00	–	–
Non-advanced DR	1.04	0.95–1.14	0.419
Advanced DR	1.21	1.10–1.34	< 0.0001
Any coronary event	1.26	1.15–1.37	< 0.0001
Any cerebrovascular event	1.14	1.03–1.26	0.009
Any comorbidity	1.63	1.51–1.75	< 0.0001

HR = hazard ratio; CI = confidence interval; PA = physical activity; HbA_1c_ = hemoglobin A_1c_; BMI = body mass index; BP = blood pressure; DKD = diabetic kidney disease; DR = diabetic retinopathy

Other factors independently associated with mortality were age, male sex, current smoking, HbA_1c_, anti-hyperglycemic (especially insulin) and anti-hypertensive treatment, other complications, particularly DKD, and comorbidities; moreover, PA level, total and HDL cholesterol, lipid-lowering treatment, and diastolic BP showed an inverse association with mortality. In contrast, triglycerides showed no significant association, whereas diabetes duration, BMI, and systolic BP did not enter the model (Table 3).

## Discussion

This analysis of the RIACE cohort provides strong evidence that having an history of diabetic foot, including ulcer/gangrene, amputation, and lower limb revascularization, markedly increases the risk of death independent of CVD risk factors, other complications and severe comorbidities.

These findings add to those of the few studies that previously showed an independent effect of foot ulcers only on mortality [20, 25, 26]. The Nord-Trøndelag Health (HUNT) 2 Study, a population-based Norwegian study including 1,494 patients with diabetes (155 with and 1,339 without an history of foot ulcer) and 63,632 nondiabetic individuals, showed that the adjusted mortality risk in those with a history of foot ulcer was more than double (+ 129%) of that of nondiabetic individuals, but only 47% higher than in those without foot ulcer [20], similar to the ~ 50% increase in the adjusted mortality risk observed in the RIACE cohort. Conversely, the analysis of data from 414,523 people with diabetes enrolled in practices associated with The Health Improvement Network in the UK showed that the adjusted risk of death was increased 2.48-fold among the 20,737 individuals who developed diabetic foot ulcers versus those who did not [26]. These data are in keeping with the greater than two-fold adjusted risk of death in a previous small-sized study in veterans of the American military services with diabetes with versus without foot ulcer [25], suggesting an even higher impact of foot ulcer per se.

The excess risk of death may be attributed to factors associated with history of diabetic foot that were not detected in the RIACE cohort and, hence, were not considered in the multivariable analyses. One of these factors may be infection complicating foot ulcer/gangrene, consistent with the findings that sepsis is one of the main causes of death after CVD [9, 24, 35] and that polymicrobial growth in deep tissue culture was found to be independently associated with mortality [36] in patients with diabetic foot. Moreover, diabetic foot may represent a marker of medical frailty [26] and is known to be associated in a bidirectional manner with depression [37, 38], which was shown to be an independent risk factor for mortality in these individuals [20, 39]. Another unmeasured confounder may be the socio-economic status, which has been shown to be a major determinant of foot ulcer development and outcomes [40, 41].

The 47% death rate in patients with any manifestation of diabetic foot, with differences according to the type(s) of event(s) (from 42% of lower limb revascularization alone to 61% of its combination with ulcer/gangrene/amputation) over a 7.42-year follow-up does not support the reported declining trend in mortality [16, 18, 19] and is rather in keeping with the study demonstrating no significant improvement [13]. The other factors independently associated with death in addition to history of diabetic foot are consistent with other studies [7, 13, 16–26] and support the concept that the presence of other micro and macrovascular complications plays a major role [7, 8], in keeping with the findings that CVD is the main cause of death among diabetic people [42] and that DKD is a major risk factor for morbidity and mortality from CVD [43].

Another finding of our study is the demonstration that, among the manifestations of diabetic foot, amputation had the greatest impact on mortality. This is consistent with previous studies showing that amputation was an independent correlate of death, together with age and albumin [17], and conferred a high mortality risk with an adjusted OR of 6.42 [22]. Likewise, a longitudinal cohort study of patients cared for in the Health Improvement Network showed a 2.37 higher adjusted mortality risk of death in those who had undergone a lower extremity amputation [44]. However, we found that mortality risk was increased in patients with amputation regardless of whether it was preceded by an ulcer/gangrene, possibly suggesting a major role for infection, which may have caused amputation in individuals without an ulcer/gangrene and, in combination with ischemia, also in some of those with an ulcer/gangrene. Conversely, the impact of lower limb revascularization was found to be relatively modest, as undergoing a revascularization procedure without having an ulcer/gangrene/amputation was associated with a ~ 23% increase in the adjusted risk of death, suggesting the importance of timely revascularization for reducing the risk of ulcer development and related mortality. Moreover, mortality risk increased only slightly for combined ulcer/gangrene/amputation and revascularization compared with ulcer/gangrene/amputation alone.

The apparently low prevalence of any diabetic foot event in the RIACE cohort (~ 6%) as compared with the reported lifetime prevalence of 19 to 34% [1] may be explained by the characteristics of the study participants. In particular, a diabetes duration of  ≤ 10 years in almost 50% of participants and the exclusion of patients on dialysis might have also contributed to such a low prevalence, which is indeed similar to the ~ 5% prevalence of foot ulcers among individuals with diabetes enrolled in the UK study mentioned above [24].

The independent correlates of history of diabetic foot event are consistent with the known risk factors for the development of foot ulcers, including CVD in the coronary or cerebrovascular beds, microvascular complications, and CVD risk factors [1, 2]. In addition, the relationship with PA level is consistent with a recent prospective study showing that sedentary behavior is significantly and independently associated with the occurrence of a diabetic foot ulcer [45].

Strength of our study include the large sample size, the completeness of baseline and follow-up data and, particularly, the assessment of a wide range of clinical parameters which allowed accounting for several confounding factors. However, there are several limitations. First, the lack of information on the causes of death did not allow detecting differences in CVD versus non-CVD deaths and the impact of other causes of death that might be associated with diabetic foot, such as infections. Second, the lack of information regarding the presence and severity of PAD (except for history of lower limb revascularization), DPN, and/or foot infection did not allow to establish the cause of foot ulcer/gangrene and amputation in the RIACE participants. Third, the lack of information on the occurrence of diabetic foot events during the follow-up may have led to an underestimation of the prevalence of this complication and of its impact on mortality. Fourth, the study findings may not be applicable to the general ambulatory population, as only part of the individuals with type 2 diabetes attend Diabetes Clinics in Italy; however, the RIACE cohort is representative of patients followed by diabetes specialists in these clinics [46]. Finally, the observational design makes causal interpretation impossible.

## Conclusions

In Caucasian patients with type 2 diabetes from the RIACE cohort, an history of diabetic foot event, including ulcer/gangrene, amputation, and lower limb revascularization, was associated with a ~ 50% increased risk of subsequent death from any cause, independent of CVD risk factors, other complications and severe comorbidities, which were also significantly associated with mortality. The association with mortality was greatest for amputation, whereas that for revascularization alone was relatively modest.

### Electronic supplementary material

Below is the link to the electronic supplementary material.

Supplementary Material 1

Supplementary Material 2

Supplementary Material 3

Supplementary Material 4

Supplementary Material 5

## Data Availability

The datasets used and/or analysed during the current study are available from the corresponding author on reasonable request.

## Declarations

### Additional file 1

The RIACE Study Group.

### Additional file 2: table S1

Baseline clinical features of study participants by history of ulcer/gangrene and/or amputation.

### Additional file 3: table S2

Baseline clinical features of study participants by history of ulcer/gangrene/amputation and/or lower limb revascularization.

### Additional file 4: table S3

Binary backward logistic regression analysis of the independent correlates of history of diabetic foot.

## Abbreviations

BMI: Body mass index
BP: Blood pressure
CI: Confidence interval
COPD: Chronic Obstructive Pulmonary Disease
CVD: Cardiovascular disease
DKD: Diabetic kidney disease
DPN: Diabetic peripheral neuropathy
DR: Diabetic retinopathy
eGFR: Estimated glomerular filtration rate
HbA_1c_: Hemoglobin A_1c_
HR: Hazard ratio
OR: Odds ratio
PA: Physical activity
PAD: Peripheral artery disease
RIACE: Renal Insufficiency and cardiovascular events
