# Supplementary material for: Independent association of history of diabetic foot with all-cause mortality in patients with type 2 diabetes: the Renal Insufficiency And Cardiovascular Events (RIACE) Italian Multicenter Study
Source: Cardiovasc Diabetol. 2024 Jan 13;23:34. doi: 10.1186/s12933-023-02107-9 (PMC10787405; doi:10.1186/s12933-023-02107-9)
Supplement: Supplementary file 2 — Supplementary Material 2 [file 12933_2023_2107_MOESM2_ESM.docx]

**Additional file 2: Table S1.** Baseline clinical features of study participants by history of ulcer/gangrene and/or amputation.

| **Variables** | **Ulcer/gangrene no Amputation no** | **Ulcer/gangrene yes Amputation no** | **Ulcer/gangrene no Amputation yes** | **Ulcer/gangrene yes Amputation yes** | ***p*** |
| --- | --- | --- | --- | --- | --- |
| **N (%)** | 15,208 (96.4) | 412 (2.6) | 33 (0.2) | 120 (0.8) |  |
| **Amputation** |  |  |  |  |  |
| **Minor** | - | - | 28 (84.8) | 101 (84.2) |  |
| **Major** | - | - | 5 (15.2) | 19 (15.8) |  |
| **Lower limb revascularization** | 330 (2.2) | 72 (17.5) | 10 (30.3) | 44 (36.7) |  |
| **Endovascular** | 123 (0.8) | 36 (8.7) | 5 (15.2) | 28 (23.3) |  |
| **Surgical** | 197 (1.3) | 34 (8.3) | 3 (9.1) | 11 (9.2) |  |
| **Both** | 10 (0.1) | 2 (0.5) | 2 (6.1) | 5 (4.2) |  |
| **Age, years** | 66.4±10.3 | 71.0±10.4 | 67.7±9.1 | 67.0±9.3 | <0.0001 |
| **Sex, n (%)** |  |  |  |  | <0.0001 |
| **Females** | 6,607 (43.4) | 166 (40.3) | 7 (21.2) | 34 (28.3) |  |
| **Males** | 8,601 (56.6) | 246 (59.7) | 26 (78.8) | 86 (71.7) |  |
| **Smoking, n (%)** |  |  |  |  | 0.002 |
| **Never** | 8,637 (56.8) | 217 (52.7) | 21 (63.6) | 53 (44.2) |  |
| **Former** | 4,239 (27.9) | 134 (32.5) | 8 (24.2) | 53 (44.2) |  |
| **Current** | 2,332 (15.3) | 61 (14.8) | 4 (12.1) | 14 (11.7) |  |
| **PA level, n (%)** |  |  |  |  | <0.0001 |
| **Inactive or moderately inactive** | 9,573 (62.9) | 342 (83.0) | 21 (63.6) | 90 (75.0) |  |
| **Moderately active** | 5,400 (35.5) | 69 (16.7) | 12 (36.4) | 30 (25.0) |  |
| **Highly active** | 235 (1.5) | 1 (0.2) | 0 (0.0) | 0 (0.0) |  |
| **Diabetes duration, years** | 13.0±10.1 | 19.2±10.9 | 16.4±9.9 | 18.8±10.5 | <0.0001 |
| **HbA_1c_, %** | 7.54±1.50 | 7.92±1.74 | 7.71±1.19 | 7.86±1.59 | <0.0001 |
| **BMI, kg·m^-2^** | 29.0±5.1 | 28.8±5.3 | 29.5±3.2 | 29.9±5.8 | 0.189 |
| **Waist circumference, cm** | 102.5±10.4 | 102.4±11.1 | 104.5±7.3 | 104.8±11.7 | 0.069 |
| **Triglycerides, mmol·l^-1^** | 1.33 (0.97-1.89) | 1.37 (0.99-1.97) | 1.29 (0.98-2.59) | 1.36 (1.07-1.83) | 0.289 |
| **Total cholesterol, mmol·l^-1^** | 4.79±0.99 | 4.63±0.99 | 4.49±1.18 | 4.37±1.06 | <0.0001 |
| **HDL cholesterol, mmol·l^-1^** | 1.29±0.35 | 1.22±0.36 | 1.17±0.34 | 1.19±0.43 | <0.0001 |
| **Non-HDL cholesterol, mmol·l^-1^** | 3.50±0.95 | 3.41±0.95 | 3.32±1.16 | 3.19±0.95 | 0.001 |
| **LDL cholesterol, mmol·l^-1^** | 2.79±0.84 | 2.70±0.86 | 2.49±0.88 | 2.49±0.89 | <0.0001 |
| **Dyslipidemia, n (%)** | 12,518 (82.3) | 321 (77.9) | 27 (81.8) | 94 (78.3) | 0.089 |
| **Systolic BP, mmHg** | 138.0±17.9 | 140.3±19.9 | 146.8±25.3 | 139.4±21.7 | 0.002 |
| **Diastolic BP, mmHg** | 78.8±9.4 | 78.5±10.0 | 78.7±11.1 | 76.2±9.7 | 0.023 |
| **Pulse pressure, mmHg** | 59.2±15.6 | 61.8±17.1 | 68.2±18.6 | 63.2±19.2 | <0.0001 |
| **Hypertension, n (%)** | 12,677 (83.4) | 368 (89.3) | 32 (97.0) | 112 (93.3) | <0.0001 |
| **Anti-hyperglycemic treatment, n (%)** |  |  |  |  | <0.0001 |
| **Lifestyle** | 2,107 (13.9) | 16 (3.9) | 1 (3.0) | 2 (1.7) |  |
| **Non-insulin** | 9,418 (61.9) | 228 (55.3) | 10 (30.3) | 25 (20.8) |  |
| **Insulin** | 3,683 (24.2) | 168 (40.8) | 22 (66.7) | 93 (77.5) |  |
| **Lipid-lowering treatment, n (%)** | 7,005 (46.1) | 193 (46.8) | 18 (54.5) | 70 (58.3) | 0.042 |
| **Anti-hypertensive treatment, n (%)** | 10,690 (70.3) | 325 (78.9) | 30 (90.9) | 104 (86.7) | <0.0001 |
| **Albuminuria, mg·day^-1^** | 13.2 (6.6-31.5) | 24.6 (11.7-94.5) | 31.6 (10.8-167.2) | 90.4 (19.3-249.6) | <0.0001 |
| **Serum creatinine, µmol·l^-1^** | 80.5±33.5 | 98.5±56.0 | 95.0±29.6 | 98.7±44.2 | <0.0001 |
| **eGFR, ml·min^-1^·1.73m^-2^** | 80.7±20.7 | 68.6±23.6 | 72.1±22.2 | 70.5±25.9 | <0.0001 |
| **DKD phenotype, n (%)** |  |  |  |  | <0.0001 |
| **No DKD** | 9,847 (64.7) | 155 (37.6) | 13 (39.4) | 32 (26.7) |  |
| **Albuminuric DKD with preserved eGFR** | 2,830 (18.6) | 110 (26.7) | 11 (33.3) | 44 (36.7) |  |
| **Nonalbuminuric DKD** | 1,420 (9.3) | 61 (14.8) | 3 (9.1) | 7 (5.8) |  |
| **Albuminuric DKD with reduced eGFR** | 1,111 (7.3) | 86 (20.9) | 6 (18.2) | 37 (30.8) |  |
| **DR, n (%)** |  |  |  |  | <0.0001 |
| **No DR** | 12,011 (79.0) | 221 (53.6) | 15 (45.5) | 29 (24.2) |  |
| **Non-advanced DR** | 1,832 (12.0) | 97 (23.5) | 6 (18.2) | 22 (18.3) |  |
| **Advanced DR** | 1,365 (9.0) | 94 (22.8) | 12 (36.4) | 69 (57.5) |  |
| **CVD, n (%)** |  |  |  |  |  |
| **Myocardial infarction** | 1,649 (10.8) | 74 (18.0) | 5 (15.2) | 30 (25.0) | <0.0001 |
| **Coronary revascularization** | 1,461 (9.6) | 89 (21.6) | 5 (15.2) | 29 (24.2) | <0.0001 |
| **Any coronary event** | 2,250 (14.8) | 122 (29.6) | 6 (18.2) | 37 (30.8) | <0.0001 |
| **Stroke** | 485 (3.2) | 17 (4.1) | 3 (9.1) | 10 (8.3) | 0.002 |
| **Carotid revascularization** | 762 (5.0) | 88 (21.4) | 2 (6.1) | 15 (12.5) | <0.0001 |
| **Any cerebrovascular event** | 1,178 (7.7) | 99 (24.0) | 5 (15.2) | 23 (19.2) | <0.0001 |
| **Any coronary or cerebrovascular event** | 2,962 (19.6) | 160 (39.3) | 10 (30.3) | 43 (37.1) | <0.0001 |
| **Comorbidities n (%)** |  |  |  |  |  |
| **Any** | 2,684 (17.6) | 87 (21.1) | 11 (33.3) | 21 (17.5) | 0.032 |
| **COPD** | 645 (4.2) | 25 (6.1) | 2 (6.1) | 6 (5.0) | 0.302 |
| **Chronic liver disease** | 1,304 (8.6) | 54 (13.1) | 5 (15.2) | 7 (5.8) | 0.004 |
| **Cancer** | 1,005 (6.6) | 18 (4.4) | 4 (12.1) | 8 (6.7) | 0.175 |

PA = physical activity; HbA_1c_ = hemoglobin A_1c_; BMI = body mass index; BP = blood pressure; eGFR = estimated glomerular filtration rate; DKD = diabetic kidney disease; DR = diabetic retinopathy; = CVD = cardiovascular disease; COPD = chronic obstructive pulmonary disease.
