# Supplementary material for: Independent association of history of diabetic foot with all-cause mortality in patients with type 2 diabetes: the Renal Insufficiency And Cardiovascular Events (RIACE) Italian Multicenter Study
Source: Cardiovasc Diabetol. 2024 Jan 13;23:34. doi: 10.1186/s12933-023-02107-9 (PMC10787405; doi:10.1186/s12933-023-02107-9)
Supplement: Supplementary file 3 — Supplementary Material 3 [file 12933_2023_2107_MOESM3_ESM.docx]

**Additional file 3: Table S2.** Baseline clinical features of study participants by history of ulcer/gangrene/amputation and/or lower limb revascularization.

| **Variables** | **Ulcer/gangr/amp no Revascularization no** | **Ulcer/gangr/amp yes Revascularization no** | **Ulcer/gangr/amp no Revascularization yes** | **Ulcer/gangr/amp yes Revascularization yes** | ***p*** |
| --- | --- | --- | --- | --- | --- |
| **N (%)** | 14,878 (94.3) | 439 (2.8) | 330 (2.1) | 126 (0.8) |  |
| **Ulcer/gangrene** | - | 416 (94.8) | - | 116 (92.1) |  |
| **Amputation** | - | 99 (22.6) | - | 54 (42.9) |  |
| **Minor** | - | 81 (18.5) | - | 48 (38.1) |  |
| **Major** | - | 18 (4.1) | - | 6 (4.8) |  |
| **Lower limb revascularization** |  |  |  |  |  |
| **Endovascular** | - | - | 123 (37.3) | 69 (54.8) |  |
| **Surgical** | - | - | 197 (59.7) | 48 (38.1) |  |
| **Both** | - | - | 10 (3.0) | 9 (7.1) |  |
| **Age, years** | 66.4±10.4 | 69.5±10.4 | 70.4±8.4 | 71.5±9.4 | <0.0001 |
| **Sex, n (%)** |  |  |  |  | <0.0001 |
| **Females** | 6,522 (43.8) | 177 (40.3) | 85 (25.8) | 30 (23.8) |  |
| **Males** | 8,356 (56.2) | 262 (59.7) | 245 (74.2) | 96 (76.2) |  |
| **Smoking, n (%)** |  |  |  |  | <0.0001 |
| **Never** | 8,511 (57.2) | 230 (52.4) | 126 (38.2) | 61 (48.4) |  |
| **Former** | 4,101 (27.6) | 155 (35.3) | 138 (41.8) | 40 (31.7) |  |
| **Current** | 2,266 (15.2) | 54 (12.3) | 66 (20.0) | 25 (19.8) |  |
| **PA level, n (%)** |  |  |  |  | <0.0001 |
| **Inactive or moderately inactive** | 9,314 (62.6) | 349 (79.5) | 259 (78.5) | 104 (82.5) |  |
| **Moderately active** | 5,333 (35.8) | 90 (20.5) | 67 (20.3) | 21 (16.7) |  |
| **Highly active** | 231 (1.6) | 0 (0.0) | 4 (1.2) | 1 (0.8) |  |
| **Diabetes duration, years** | 12.8±10.0 | 19.0±10.9 | 19.5±10.4 | 18.8±10.5 | <0.0001 |
| **HbA_1c_, %** | 7.53±1.50 | 7.97±1.76 | 7.97±1.42 | 7.62±1.36 | <0.0001 |
| **BMI, kg·m^-2^** | 29.0±5.2 | 29.5±5.6 | 28.3±4.3 | 27.8±4.0 | 0.001 |
| **Waist circumference, cm** | 102.5±10.4 | 103.7±11.6 | 101.6±9.1 | 100.6±8.8 | 0.008 |
| **Triglycerides, mmol·l^-1^** | 1.33 (0.96-1.88) | 1.37 (1.00-1.97) | 1.47 (1.03-2.10) | 1.32 (1.03-2.00) | 0.083 |
| **Total cholesterol, mmol·l^-1^** | 4.80±0.99 | 4.62±0.99 | 4.56±0.98 | 4.40±1.12 | <0.0001 |
| **HDL cholesterol, mmol·l^-1^** | 1.30±0.35 | 1.21±0.37 | 1.18±0.32 | 1.20±0.40 | <0.0001 |
| **Non-HDL cholesterol, mmol·l^-1^** | 3.50±0.95 | 3.40±0.94 | 3.37±0.91 | 3.20±1.05 | <0.0001 |
| **LDL cholesterol, mmol·l^-1^** | 2.80±0.84 | 2.68±0.86 | 2.61±0.81 | 2.49±0.90 | <0.0001 |
| **Dyslipidemia, n (%)** | 12,214 (82.1) | 339 (77.2) | 304 (92.1) | 103 (81.7) | <0.0001 |
| **Systolic BP, mmHg** | 138.0±17.9 | 141.0±20.4 | 137.2±17.8 | 138.5±21.5 | 0.005 |
| **Diastolic BP, mmHg** | 78.8±9.4 | 78.6±10.0 | 76.3±9.5 | 75.8±10.1 | <0.0001 |
| **Pulse pressure, mmHg** | 59.1±15.6 | 62.4±17.6 | 60.9±15.4 | 62.7±18.3 | <0.0001 |
| **Hypertension, n (%)** | 12,369 (83.1) | 394 (89.7) | 308 (93.3) | 118 (93.7) | <0.0001 |
| **Anti-hyperglycemic treatment, n (%)** |  |  |  |  | <0.0001 |
| **Lifestyle** | 2,076 (14.0) | 12 (2.7) | 31 (9.4) | 7 (5.6) |  |
| **Non-insulin** | 9,229 (62.0) | 215 (49.0) | 189 (57.3) | 48 (38.1) |  |
| **Insulin** | 3,573 (24.0) | 212 (48.3) | 110 (33.3) | 71 (56.3) |  |
| **Lipid-lowering treatment, n (%)** | 6,746 (45.3) | 196 (44.6) | 259 (78.5) | 85 (67.5) | <0.0001 |
| **Anti-hypertensive treatment, n (%)** | 10,398 (69.9) | 346 (78.8) | 292 (88.5) | 113 (89.7) | <0.0001 |
| **Albuminuria, mg·day^-1^** | 13.1 (6.5-31.0) | 26.0 (12.0-112.1) | 17.4 (8.1-57.2) | 57.1 (19.7-210.7) | <0.0001 |
| **Serum creatinine, µmol·l^-1^** | 80.1±33.3 | 96.3±54.4 | 95.8±36.5 | 105.5±44.3 | <0.0001 |
| **eGFR, ml·min^-1^·1.73m^-2^** | 80.9±20.7 | 70.6±24.2 | 69.8±20.2 | 64.4±22.8 | <0.0001 |
| **DKD phenotype, n (%)** |  |  |  |  | <0.0001 |
| **No DKD** | 9,697 (65.2) | 169 (38.5) | 150 (45.5) | 31 (24.6) |  |
| **Albuminuric DKD with preserved eGFR** | 2,767 (18.6) | 125 (28.5) | 63 (19.1) | 40 (31.7) |  |
| **Nonalbuminuric DKD** | 1,366 (9.2) | 56 (12.8) | 54 (16.4) | 15 (11.9) |  |
| **Albuminuric DKD with reduced eGFR** | 1,048 (7.0) | 89 (20.3) | 63 (19.1) | 40 (31.7) |  |
| **DR, n (%)** |  |  |  |  | <0.0001 |
| **No DR** | 11,797 (79.3) | 224 (51.0) | 214 (64.8) | 41 (32.5) |  |
| **Non-advanced DR** | 1,758 (11.8) | 95 (21.6) | 74 (22.4) | 30 (23.8) |  |
| **Advanced DR** | 1,323 (8.9) | 120 (27.3) | 42 (12.7) | 55 (43.7) |  |
| **CVD, n (%)** |  |  |  |  |  |
| **Myocardial infarction** | 1,503 (10.1) | 68 (15.5) | 146 (44.2) | 41 (32.5) | <0.0001 |
| **Coronary revascularization** | 1,246 (8.4) | 65 (14.8) | 215 (65.2) | 58 (46.0) | <0.0001 |
| **Any coronary event** | 2,020 (13.6) | 97 (22.1) | 230 (69.7) | 68 (54.0) | <0.0001 |
| **Stroke** | 463 (3.1) | 19 (4.3) | 22 (6.7) | 11 (8.7) | <0.0001 |
| **Carotid revascularization** | 589 (4.0) | 55 (12.5) | 173 (52.4) | 50 (39.7) | <0.0001 |
| **Any cerebrovascular event** | 1,001 (6.7) | 71 (16.2) | 177 (53.6) | 56 (44.4) | <0.0001 |
| **Any coronary or cerebrovascular event** | 2,715 (18.4) | 139 (32.1) | 247 (75.5) | 74 (60.2) | <0.0001 |
| **Comorbidities n (%)** |  |  |  |  |  |
| **Any** | 2,614 (17.6) | 92 (21.0) | 70 (21.2) | 27 (21.4) | 0.063 |
| **COPD** | 621 (4.2) | 25 (5.7) | 24 (7.3) | 8 (6.3) | 0.012 |
| **Chronic liver disease** | 1,264 (8.5) | 52 (11.8) | 40 (12.1) | 14 (11.1) | 0.007 |
| **Cancer** | 990 (6.7) | 23 (5.2) | 15 (4.5) | 7 (5.6) | 0.277 |

PA = physical activity; HbA_1c_ = hemoglobin A_1c_; BMI = body mass index; BP = blood pressure; eGFR = estimated glomerular filtration rate; DKD = diabetic kidney disease; DR = diabetic retinopathy; = CVD = cardiovascular disease; COPD = chronic obstructive pulmonary disease.
