# Supplementary material for: Independent association of history of diabetic foot with all-cause mortality in patients with type 2 diabetes: the Renal Insufficiency And Cardiovascular Events (RIACE) Italian Multicenter Study
Source: Cardiovasc Diabetol. 2024 Jan 13;23:34. doi: 10.1186/s12933-023-02107-9 (PMC10787405; doi:10.1186/s12933-023-02107-9)
Supplement: Supplementary file 4 — Supplementary Material 4 [file 12933_2023_2107_MOESM4_ESM.docx]

**Additional file 4: Table S3.** Binary non-conditional multivariable logistic regression analysis with backward stepwise selection of variables of the independent correlates of history of diabetic foot.

| **Variables** | **Beta** | **Standard error** | ***p*** |
| --- | --- | --- | --- |
| **Age, years** | 1.01 | 1.00-1.02 | 0.038 |
| **Male sex** | 1.26 | 1.06-1.49 | 0.008 |
| **Smoking status** |  |  | <0.0001 |
| **Never** | 1.00 |  |  |
| **Former** | 1.39 | 1.18-1.65 | <0.0001 |
| **Current** | 1.38 | 1.11-1.72 | 0.003 |
| **PA level** |  |  | <0.0001 |
| **Inactive or moderately inactive** | 1.00 |  |  |
| **Moderately active** | 0.53 | 0.44-0.63 | <0.0001 |
| **Highly active** | 0.36 | 0.14-0.93 | 0.034 |
| **Diabetes duration, years** | 1.02 | 1.01-1.03 | <0.0001 |
| **BMI, kg·m^-2^** | 0.99 | 0.97-1.00 | 0.083 |
| **HDL cholesterol, mmol·l^-1^** | 0.99 | 0.98-0.99 | <0.0001 |
| **Anti-hyperglycemic treatment** |  |  | <0.0001 |
| **Lifestyle** | 1.00 |  |  |
| **Non-insulin** | 1.37 | 1.00-1.88 | 0.050 |
| **Insulin** | 1.95 | 1.41-2.70 | <0.0001 |
| **Lipid-lowering treatment** | 1.18 | 1.01-1.37 | 0.038 |
| **DKD phenotype** |  |  | <0.0001 |
| **No DKD** | 1.00 |  |  |
| **Albuminuric DKD with preserved eGFR** | 1.53 | 1.27-1.85 | <0.0001 |
| **Nonalbuminuric DKD** | 1.44 | 1.14-1.84 | 0.003 |
| **Albuminuric DKD with reduced eGFR** | 1.91 | 1.53-2.38 | <0.0001 |
| **DR grade** |  |  | <0.0001 |
| **No DR** | 1.00 |  |  |
| **Non-advanced DR** | 1.68 | 1.38-2.03 | <0.0001 |
| **Advanced DR** | 2.56 | 2.10-3.12 | <0.0001 |
| **Any coronary event** | 2.68 | 2.28-3.15 | <0.0001 |
| **Any cerebrovascular event** | 3.61 | 3.04-4.29 | <0.0001 |

HbA_1c_, triglycerides, total cholesterol, systolic and diastolic BP, anti-hypertensive treatment, and any comorbidity were excluded from the model. OR = odds ratio; CI = confidence interval; PA = physical activity; HbA_1c_ = hemoglobin A_1c_; BMI = body mass index; BP = blood pressure; DKD = diabetic kidney disease; DR = diabetic retinopathy.
